# STDP encodes oscillation frequencies in the connections of recurrent networks of spiking neurons

**DOI:** 10.1186/1471-2202-13-S1-P130

**Published:** 2012-07-16

**Authors:** Robert R Kerr, Anthony N Burkitt, Doreen A Thomas, David B Grayden

**Affiliations:** 1 NeuroEngineering Lab, Dept. Electrical & Electronic Engineering, University of Melbourne, VIC 3010, Australia; 2 Centre for Neural Engineering, University of Melbourne, VIC 3010, Australia; 3 Dept. of Mechanical Engineering, University of Melbourne, VIC 3010, Australia; 4 Bionics Institute, 384 Albert St., East Melbourne, VIC 3002, Australia

## 

Spike-timing-dependent plasticity (STDP) is a learning rule that updates synaptic strengths based on the relative timing of pre- and post-synaptic spikes. Unlike rate-based Hebbian learning, STDP can potentially encode fast temporal correlations in neuronal activity, such as oscillations, in the functional structure of networks of neurons that have axonal and dendritic propagation delays. The motivation behind this study was to understand the different ways that spatiotemporal patterns can be learnt by the recurrent connections in a network of neurons with STDP present. This understanding is vital to uncovering the mechanisms by which basic learning and information processing tasks are performed throughout the brain. A specific example in which these mechanisms may contribute is in explaining how the brain can perceive the pitch of complex sounds up to 300Hz. This work employs and builds upon the analytical framework for learning with STDP used in a previous study [1].

**Figure 1:**
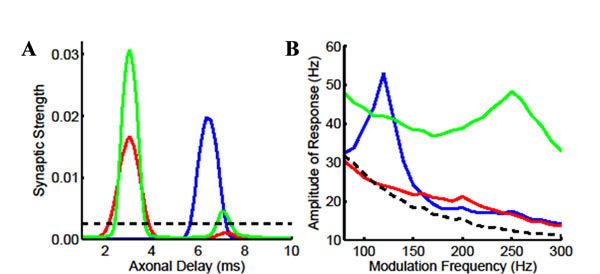
Results of simulations with networks of 10,000 LIF neurons. A: The mean synaptic weight with axonal delay of a network before learning (dashed: all weights initially set to 0.0025 with an even spread of delays over 1-10ms) and after learning for 20,000s with STDP (blue: ‘medium’ dynamics and 120Hz inputs; red: ‘medium’ dynamics and 240Hz inputs; and green: ‘fast’ dynamics and 240Hz inputs). B: The frequency-response plot with the average maximum of the oscillatory response of the networks in A. Note: ‘medium/fast’ dynamics correspond to 10ms/5ms membrane time constant, 0.5ms/0.1ms synaptic rise time, and 1ms/0.5ms synaptic decay time. These are realistic for neurons/synapses found in the auditory brainstem.

In this study, the changes made by additive STDP to synaptic strengths in recurrent networks with axonal delays receiving oscillatory inputs were investigated analytically with the Poisson neuron model and verified through simulations with leaky integrate-and-fire (LIF) neurons. Frequencies between 100-300Hz were considered, which correspond to the modulation frequencies found in the auditory brainstem representing the fundamental frequency of different natural sounds. The analysis and simulations found that connections were selectively potentiated and depressed based on their axonal delay in such a way that the delays of the strong connections in the network “resonated” with the input frequency (Figure 1A). The trained networks were found to respond selectively to the frequency they were trained with (Figure 1B). Higher frequencies (e.g. 240Hz as shown here) would always be learnt by the network, but in order to show a selective response after learning they needed faster neuronal and synaptic time constants (see details in Figure 1 caption).
